# Brain MRI Findings of Hemophagocytic Lymphohistiocytosis With a Heterozygous PRF1 Gene Mutation Masquerading As CLIPPERS: A Case Report

**DOI:** 10.7759/cureus.36787

**Published:** 2023-03-28

**Authors:** Arisa Tachibana, Ryo Kurokawa, Aristides A Capizzano, David N Irani, Mariko Kurokawa, Akira Baba, John Kim, Toshio Moritani

**Affiliations:** 1 Radiology, University of Michigan, Ann Arbor, USA; 2 Radiology, The University of Tokyo, Tokyo, JPN; 3 Neurology, University of Michigan, Ann Arbor, USA

**Keywords:** neuroimaging, gene mutation, prf1, chronic lymphocytic inflammation with pontine perivascular enhancement responsive to steroids, hemophagocytic lymphohistiocytosis

## Abstract

Familial hemophagocytic lymphohistiocytosis is a rare and potentially life-threatening genetic condition characterized by unsuppressed immune activation and hypercytokinemia. Chronic Lymphocytic Inflammation with Pontine Perivascular Enhancement Responsive to Steroids (CLIPPERS) is a central nervous system inflammatory disorder characterized by punctate and curvilinear gadolinium-enhancing lesions in the brainstem, cerebellum, and spinal cord, which responds well to corticosteroid treatment. Hemophagocytic lymphohistiocytosis has been known to mimic CLIPPERS on neuroimaging, and patients previously diagnosed with CLIPPERS may carry familial hemophagocytic lymphohistiocytosis-related gene mutations that serve as predisposing factors. In this article, we describe a case initially diagnosed with CLIPPERS based on characteristic magnetic resonance imaging features and clinical course, who was later diagnosed with hemophagocytic lymphohistiocytosis based on a heterozygous familial hemophagocytic lymphohistiocytosis-associated *PRF1* gene mutation.

## Introduction

Familial hemophagocytic lymphohistiocytosis (FHL) is a rare hereditary disorder of the immune system characterized by unsuppressed leukocyte activation and hypercytokinemia [1]. Chronic Lymphocytic Inflammation with Pontine Perivascular Enhancement Responsive to Steroids (CLIPPERS) is an inflammatory disorder characterized by the appearance of punctate and curvilinear gadolinium-enhancing lesions in the pons and the cerebellum on magnetic resonance imaging (MRI) [2]. Recently, it has been found that patients diagnosed with CLIPPERS occasionally carry genetic mutations associated with FHL [3]. However, the MRI features in these patients remain poorly characterized. In this report, we describe a patient initially diagnosed with CLIPPERS based on characteristic clinical and MRI findings who was later diagnosed with hemophagocytic lymphohistiocytosis (HLH) based on a heterozygous FHL-related *PRF1 *gene mutation.

## Case presentation

**Figure 1:**
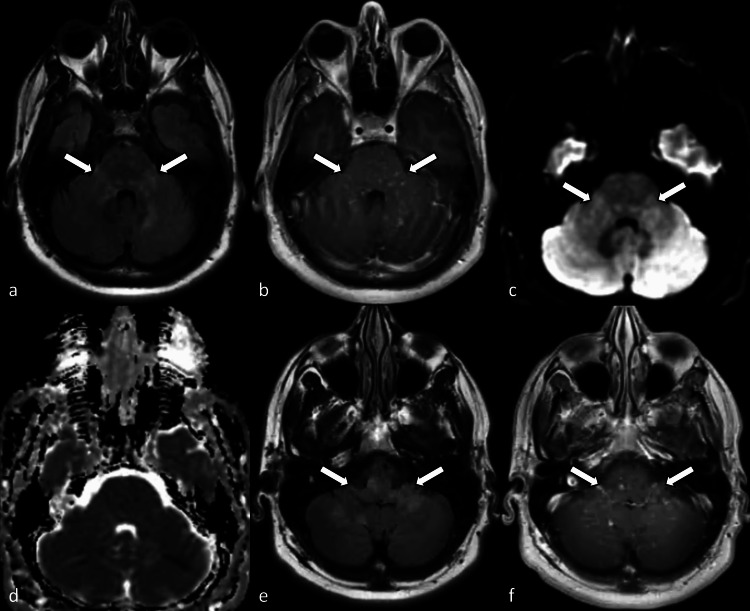
Brain MRI a, e: Fluid-attenuated inversion recovery (FLAIR) axial images, b, f: contrast-enhanced T1-weighted axial images, c: diffusion-weighted axial image, d: apparent diffusion coefficient map. Brain MRI shows multiple nodular FLAIR hyperintensity in the pons, bilateral middle cerebellar peduncles, cerebellar hemispheres, and medullar oblongata with corresponding contrast enhancement and diffusion-weighted hyperintensity without definite diffusion restriction (a-f, arrows).

A 45-year-old previously healthy man first presented with worsening double vision in 2012. By the time he came to medical attention, his right eye was unable to move within the orbit. His family history included arthritis in his father and myocardial infarction in his mother. He was a non-smoker, did not consume alcohol, and did not use any illicit drugs. Brain MRI revealed multiple hyperintense foci with contrast enhancement in the pons, bilateral middle cerebellar peduncles, and cerebellar hemispheres (Figure 1).

The characteristic MRI findings suggested CLIPPERS, and in keeping with the diagnosis, the patient’s neurologic symptoms and MRI abnormalities proved to be remarkably responsive to corticosteroid therapy over time. Indeed, when azathioprine was added to his regimen and prednisone slowly weaned, he developed recurrent double vision, malaise, and imbalance over the next 6 months that abated with the resumption of the steroids. With the resumption of the steroids, the patient had no loss of vision, no difficulty with chewing, swallowing, talking, walking, balance, or coordination, no weakness in his extremities, no numbness or tingling except numbness in his right great toe, no bowel or bladder dysfunction, or seizures. He remained stable on at least 25 mg of prednisone daily. Approximately 8 years later, the patient developed periodic fevers of unknown origin. Extensive workup for an underlying connective tissue disorder was unremarkable. His SARS-CoV-2 polymerase chain reaction results were negative. Laboratory findings are summarized in Table 1.

**Table 1:** Laboratory data WBC, white blood cell; RBC, red blood cell; IU, international unit

Laboratory parameters	Value	Reference range and units
WBC count	0.7	3.8–10.6 ×10^3^/µL
RBC count	3.09	4.40–6.00 M/µL
Hemoglobin	10.5	13.5–17.0 g/dL
Hematocrit	31.4	41–53 %
Platelet count	85	150–450 ×10^3^/µL
Ferritin	2,734	24–336 ng/mL
Aspartate aminotransferase	99	<35 IU/L
Alanine aminotransferase	71	<52 IU/L
Triglyceride	349	40–200 mg/dL

Elevated levels of ferritin, aspartate aminotransferase, alanine aminotransferase, and triglyceride, and pancytopenia were found. He underwent a bone marrow biopsy, which showed hypocellular marrow without any evidence of a neoplasm. When the patient was hospitalized the following month because of another fever spike, a massively elevated serum ferritin level of 18,356 ng/mL was found. A second bone marrow biopsy showed evidence of hemophagocytosis, and a diagnosis of HLH was made. Ultimately, the patient underwent successful bone marrow transplantation, and follow-up brain MRI examinations were normal. After transplantation, the patient reported gradual improvement in his overall stamina and sense of well-being without opportunistic infection or worsening of his neurological symptoms. The patient denied any ongoing vision or balance issues. His feet continued to burn, but gabapentin taken at night helped. The patient denied recent or ongoing complaints related to hearing, speech, chewing, swallowing, dexterity, strength, sensation, balance, mood, memory, concentration, or bowel or bladder function. A follow-up brain MRI showed improvement in the abnormal imaging findings. His HLH genetic panel results revealed a heterozygous mutation in the *PFR1* gene (PRF1 c.853_855del).

## Discussion

In this report, we describe a patient who was initially diagnosed with CLIPPERS based on characteristic MRI features and clinical course, who multiple years later was found to have HLH with a heterozygous *PRF1* gene mutation.

HLH is a rare and potentially life-threatening condition affecting the immune system. HLH occurs when immune cells such as macrophages, cytotoxic T cells, and natural killer cells, become overactive and attack healthy cells in the body, including blood cells ("hemophagocytosis") and organs [4]. HLH can be classified into two main types: familial HLH (also known as primary or genetic HLH or FHL) and secondary HLH. FHL is a genetic disorder caused by mutations in genes related to the immune system, including *PRF1*, *UNC13D*, *STX11*, and *STXBP2* associated with familial early-onset HLH [3]. Secondary HLH is more common and occurs as a complication of other underlying medical conditions such as viral infections (e.g., Epstein-Barr virus, cytomegalovirus, or human immunodeficiency virus), autoimmune diseases (e.g., systemic lupus erythematosus and juvenile idiopathic arthritis), and malignancies (e.g., lymphoma and leukemia) [5,6]. The diagnosis of HLH is established when one of the following criteria is fulfilled: (1) a molecular diagnosis consistent with HLH or (2) five of the following eight criteria are met: fever, splenomegaly, cytopenia affecting at least two of three lineages in the peripheral blood, hypertriglyceridemia and/or hypofibrinogenemia, hemophagocytosis, low or absent NK-cell activity, hyperferritinemia, and high levels of soluble interleukin-2 receptor [7]. FHL is inherited in an autosomal recessive form, and its incidence has been estimated as 0.12-0.34 per 100,000 population [1]. Most patients with FHL are diagnosed in their first year of life, whereas secondary HLH mainly affects adults (mean age approximately 50 years) [8]. Interestingly, approximately 14% of adult patients with secondary HLH have allelic abnormalities associated with FHL, meaning these polymorphisms may serve as predisposing factors [8].

Brain MRI findings of HLH can be classified into three types: multifocal T2-weighted/FLAIR (fluid-attenuated inversion recovery) hyperintensity with contrast enhancement and microhemorrhage in the brain; CLIPPERS-like pontine and cerebellar nodular/patchy T2-weighted/FLAIR hyperintensity with contrast enhancement; and leptomeningeal/cranial nerves involvement [9-11]. Inflammatory, infectious, demyelinating, and neoplastic disorders can be considered as alternative diagnoses. Differences in the brain MRI findings between FHL and secondary HLH have not yet been established.

CLIPPERS is a central nervous system inflammatory disorder characterized by punctate and curvilinear gadolinium-enhancing lesions in the brain stem, cerebellum, and spinal cord, and responds well to corticosteroid treatment [2]. The proposed diagnostic criteria for CLIPPERS include clinical, MRI, and neuropathological (i.e., perivascular and parenchymal central nervous system (CNS)infiltrates composed of T cells and macrophages) findings [2], and patients fulfilling all clinical, radiological, and neuropathological criteria are defined as having definite CLIPPERS, while those fulfilling all clinical and radiological criteria without neuropathological examination are defined as having probable CLIPPERS. The MRI features of CLIPPERS include homogenous hyperintense areas (<3 mm in diameter) on T2-weighted imaging with contrast enhancement, without ring enhancement or mass effects, predominantly in the pons and cerebellum [2]. The MRI findings in the present case were consistent with CLIPPERS. However, the lack of neuropathological confirmation in most cases introduces diagnostic uncertainty. Taieb et al. reported three patients with biallelic *PRF1* mutations and one patient with biallelic *UNC13D* mutations among 12 definite or probable CLIPPERS cases [3]. Benson et al. reported that one of four pediatric patients with CNS-isolated inflammation associated with FHL gene mutations showed CLIPPERS-like brain MRI features [10]. These studies indicate that patients diagnosed with CLIPPERS may also have FHL or HLH with FHL-associated gene mutations.

## Conclusions

HLH occasionally masquerades as CLIPPERS on MRI, and genetic mutations associated with FHL may serve as a predisposing factor for the development of CLIPPERS. Considering that hematopoietic stem cell transplantation may be curative in FHL, it is imperative to accurately differentiate HLH with FHL-associated gene mutations from CLIPPERS.
